# Development of resveratrol with thiolated alginate as a supplement to prevent nonalcoholic fatty liver disease (NAFLD)

**DOI:** 10.1063/5.0081695

**Published:** 2022-02-08

**Authors:** Yong-Chen Ke, Tzu-Chien Chen, Rui-Chian Tang, Jhih-Ni Lin, Feng-Huei Lin

**Affiliations:** 1 Department of Biomedical Engineering, College of Medicine and College of Engineering, National Taiwan University, No. 49, Fanglan Rd., Taipei 10672, Taiwan; 2 Institute of Biomedical Engineering and Nanomedicine, National Health Research Institutes, No. 35, Keyan Rd., Zhunan, Miaoli County 35053, Taiwan

## Abstract

Nowadays, nonalcoholic fatty liver disease is a common metabolic liver disease of all ages worldwide. However, current pharmacological and surgical treatments are accompanied with side effects and complications. EndoBarrier, a less invasive bariatric surgery, blocks the upper portion of the intestine to reduce nutrition absorption. To mimic the nutrient restriction effect of EndoBarrier, thiol-containing materials may bind to the thiol groups of the mucus with an enhanced mucoadhesive property. Here, we develop thiolated alginate with cysteine conjugation via an N-(3-dimethylaminopropyl)-N-ethylcarbodiimide/N-hydroxysuccinimide reaction. The alginate–cysteine (AC) exhibits excellent mucoadhesive properties and forms a physical barrier in the intestine to reduce absorption significantly, which was tested with both *in vitro* and *in vivo* mucoadhesive test and barrier function test. The nontoxicity property of AC was also proven with WST-1 and live and dead stain. In addition, AC demonstrates potent carrier properties of extending the release of resveratrol to improve the efficacy with the test of the transwell system in the release profile. In the long-term therapeutic evaluation, alginate cysteine with resveratrol (ACR) is orally administrated daily to mice with an methionine choline-deficient diet. The results of this *in vivo* study show that developed ACR could effectively alleviate fat degeneration in the liver and improve fat-related metabolic parameters in serum without hepatocellular damage and kidney dysfunction. In sum, AC was found to be mucoadhesive, reduce glucose absorption, alleviate inflammation, and decrease fatty degradation. This promising material exhibits the potential to be a supplement for nonalcoholic fatty liver disease.

## I. INTRODUCTION

Nonalcoholic fatty liver disease (NAFLD) is a chronic progressive liver disease, developing from simple steatosis, nonalcoholic steatohepatitis (NASH), cirrhosis, and finally hepatocellular carcinoma. NAFLD is categorized into a series of diseases including nonalcoholic fatty liver and nonalcoholic steatohepatitis in the spectrum of liver damage.^1^ It is defined as more than 5% of fat accumulation in the liver in the absence of competing, chronic liver disease or alcohol consumption.^2^ The epidemic of NAFLD is believed to be highly relevant to unhealthy lifestyle, genetic factors,^3^ and metabolic syndromes, such as type II diabetes, dyslipidemia, and insulin resistance.^4^ As reported earlier, the prevalence of NAFLD in overweight and diabetic individuals is about 70% and is even up to 90% in morbidly obese individuals.^5^ Since the symptoms that happen in the early stages are not noticeable with 20%–30% of the possibility for the patient with steatosis to progress into steatohepatitis,^6^ NAFLD is considered as a major threat and is in urgent need to solve. In this study, we focus on the early stage of NAFLD progress to prevent irreversible damage to the liver.

Since the disease highly correlates with excessive nutrient uptake, common beneficial treatments for NAFLD progression are lifestyle intervention, drugs, and bariatric surgery. It is suggested that lifestyle intervention is an effective way to improve NAFLD, including energy restriction and health supplement intake. Through lifestyle modification to reduce excessive intake of the substrate, nonalcoholic steatohepatitis (NASH) progression can be prevented or reversed.^7^ However, patients' low adherence rate has been a major problem in clinical practices. For pharmaceutical treatment, insulin-sensitizing agents, hepatoprotective agents, or other fat metabolism-interfering compounds are another choice, yet they all suffer from various side effects.^8^ Bariatric surgeries providing rapid weight loss is also recommended for obese patients.^9^ Although showing more remarkable effects in the short term, they accompany higher risks and surgery-related complications. EndoBarrier is an endoscopically administered duodenum polymer sleeve anchored on the pylorus that blocks the major digestion parts to reduce nutrition absorption in the intestine. Consequently, it achieves improvements in metabolic factors in a less invasive way. However, the device needs secondary surgery to remove and possesses the crisis of gastrointestinal hemorrhage.^10^ Therefore, we aim to develop an orally administered mucoadhesive material that mimics the blocking effects of EndoBarrier. Furthermore, utilizing the material's mucoadhesive property, the material may serve as a carrier to allow drugs to remain in the intestine for a longer time and enhance the bioavailability.

Alginate is a mucoadhesive natural polysaccharide extracted from algae, which is widely used in the food industry due to its gelling and stabilizing properties.^11^ It is also indicated to promote weight loss and anti-inflammation effects, decrease the permeability of intestinal mucus, and improve steatosis and metabolic disorders with alginate supplements, which showed its potential to solve the health issue as a dietary supplement.^12–15^ However, it remains in the gastrointestinal tracts for a relatively short amount of time due to the constant peristalsis and excretion. In addition, the material which formed intestinal coating has been proved to decrease the nutrition uptake.^16^ Therefore, we aim to engineer the chemical properties of alginate for a longer retention time by investigating the microenvironment where the material attaches the mucus layer. The mucus layer mostly consists of mucin, a kind of glycoprotein with a free thiol group for oxidation–reduction reaction.^17^ As materials with the thiol group interact with the mucus, they form disulfide bonding spontaneously.^18^ Therefore, we hypothesized that by thiol group conjugation, the mucoadhesive properties of alginate could be greatly enhanced, forming a mucus adhesive intestinal coating and, thus, reducing excessive nutrition absorption. In this study, a thiol-containing amino acid, cysteine, was used to modify alginate. Furthermore, we hypothesize that thiolated alginate could be a drug carrier given its prolonged retention time *in vivo*.

Previous studies have proved that a wide variety of herbs have the potential for NAFLD therapy.^19–21^ Among all, resveratrol is a natural polyphenol, generally found in grapes, blueberries, peanuts, and traditional Chinese herbal, which demonstrated several beneficial effects, such as being anti-inflammatory, antioxidative, and protective of aging, cancer, and cardiovascular diseases.^22–26^ It also serves as a supplement to decrease hepatic steatosis and inflammation. However, reports have indicated the limitation of resveratrol for its poor bioavailability attributed to the rapid metabolism.^27^ To solve this issue, we believe that the combination of resveratrol with our drug carrier may extend its release time, improving the bioavailability by the increased retention time of thiolated alginate.

**FIG. 1.**
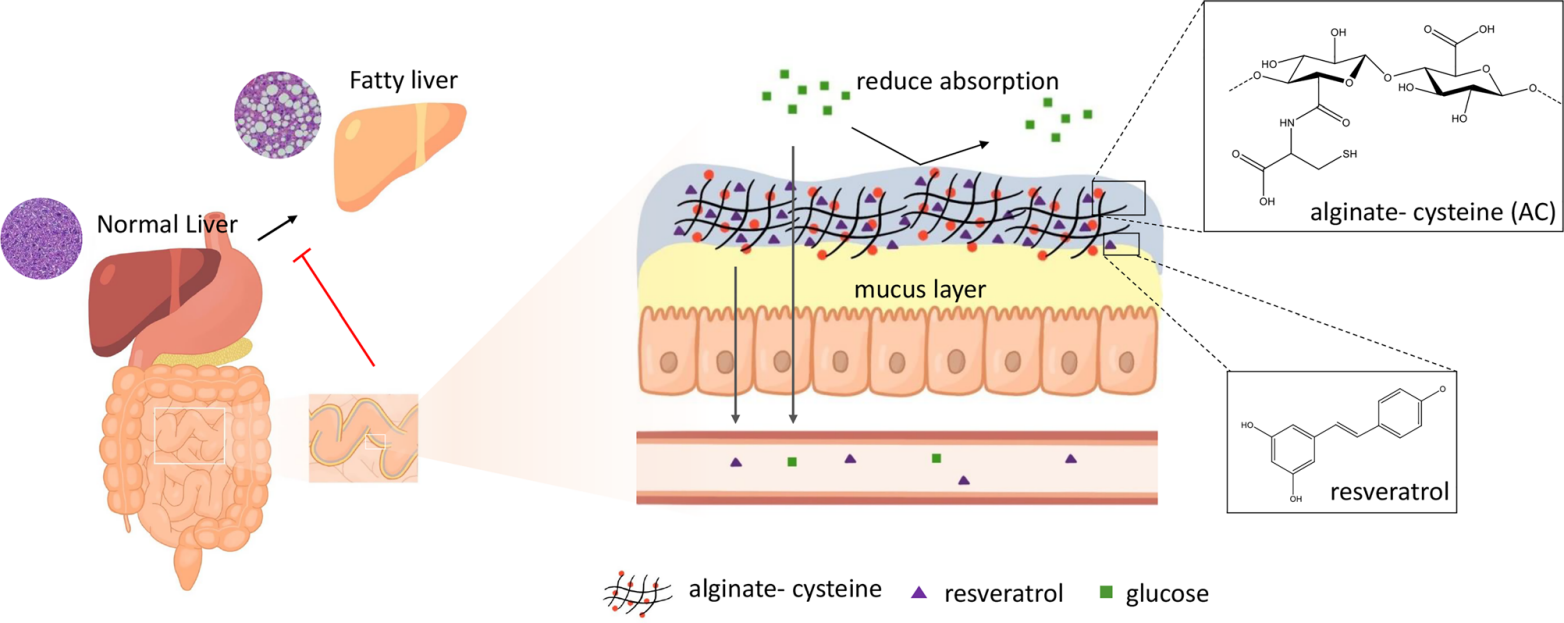
Schematic illustration of the study. The synthesized AC with resveratrol encapsulation (ACR) was utilized as a dietary supplement to attenuate NAFLD with the effect of energy restriction and controlled release of drugs.

Accordingly, we combine the idea of energy restriction by mimicking EndoBarrier and drug carrier for the delivery of resveratrol as a food supplement (Fig. 1). The barrier function of the polymer synergizes with the effect of resveratrol to alleviate NAFLD. In this study, EDC/NHS was used to cross-link alginate and cysteine (AC). The obtained AC served as a mucoadhesive material, forming disulfide bonding with intestinal mucus and formed a physical barrier. In addition, resveratrol was capsuled in AC (ACR) to extend the release time and applied in the long-term therapeutic evaluation of NAFLD. The developed AC was characterized to ensure its chemical structure and the degree of substitution. Aside from this, the barrier function, mucoadhesive property, and cytotoxicity were investigated. Finally, the therapeutic effect of ACR on alleviating NAFLD was evaluated *in vivo*.

## II. RESULTS

### A. Characterization of AC

**FIG. 2.**
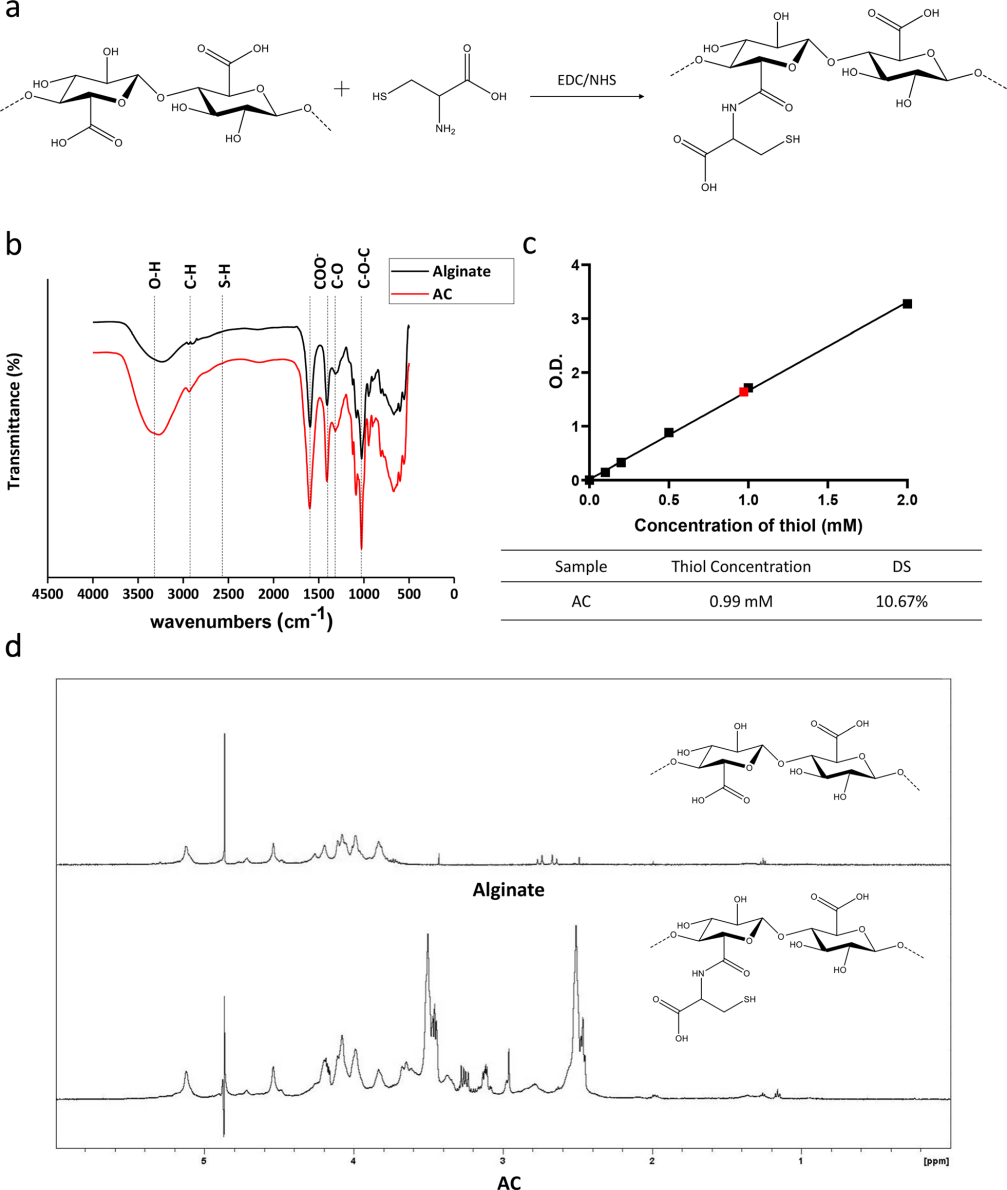
Identification of the functional groups and chemical structure of alginate and AC. (a) Schematic illustration of alginate–cysteine conjugation through EDC/NHS. (b) FTIR spectrum, the shoulder between 2550 and 2660 cm^−1^ in the spectrum was attributed to the thiol group from cysteine. (c) Evaluation of the degree of substitution of the thiol group with Ellman's assay. The concentration of the thiol group in AC was 0.99 mM, and the degree of substitution was 10.67%. (d) 1H-NMR spectrum, typical peaks of alginate showed in both spectroscopy results, and the additional peaks showed at 3.2 and 4.2 ppm in AC spectrum were attributed to cysteine.

The covalent linkage of cysteine to alginate was achieved and evaluated [Fig. 2(a)]. FTIR was used to identify the respective functional groups of AC and alginate [Fig. 2(b)]. The typical absorption band of alginate, mainly the O–H stretching at 3250 cm^−1^, pyranoid ring C–H stretching at 2901 and 2942 cm^−1^, COO^-^ symmetric stretching at 1595 cm^−1^, asymmetric stretching at 1408 cm^−1^, C–O stretching at 1316 cm^−1^, and C–O–C stretching at 1025 cm^−1^^28^ were present. As for the spectrum of AC, an additional characteristic peak from 2550 to 2660 cm^−1^ was found to be the thiol group. However, we hypothesized that it is due to the influence of –OH stretching vibration in the carboxyl group of alginate.

1H-NMR analysis was carried out to further confirm the conjugation of thiol to alginate [Fig. 2(d)]. The multiplet between 3.5 and 5.2 ppm was attributed to methine protons of hexuronic acid residue from the alginate.^29^ In the AC spectrum, typical peaks of the protons from alginate were found, and the additional peaks at 3.2 and 4.2 ppm were attributed to the proton in cysteine, indicating that cysteine was successfully conjugated with alginate.

The surface morphology of alginate and AC was studied by SEM, and the representative images are shown in Fig. S1. The SEM photographs of AC showed smoothness after modification and dialysis. The results of the elemental analysis of alginate and AC were shown in Table S1. Since alginate did not have any functional group containing sulfur, the results were normalized to alginate to eliminate the contamination of sulfur-containing compounds in the air. EDS analysis of AC showed 2.15% of sulfur and therefore revealed the attachment of cysteine. The degree of sulfur substitution was found to be 10% using Ellman's assay [Fig. 2(c)].

### B. Cell viability and cytotoxicity

**FIG. 3.**
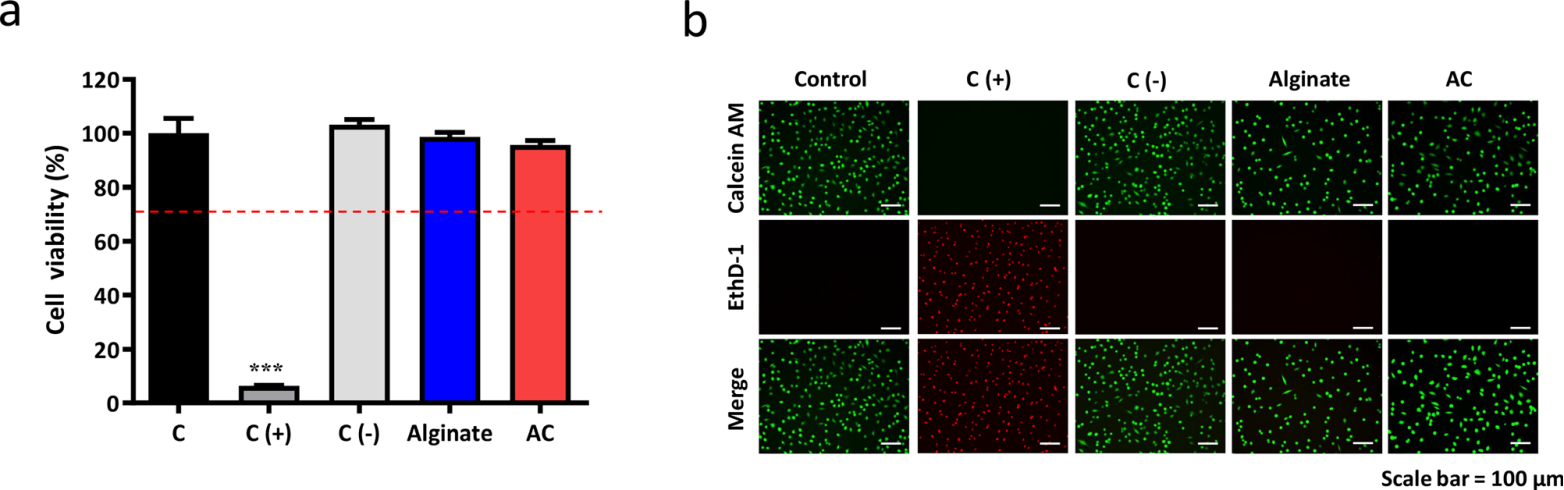
Cell viability and cytotoxicity assay. (a) Cell viability evaluation of alginate and AC via WST-1 assay (n = 6, ^***^P < 0.001 compared to the control). Zinc diethyldithiocarbamate and aluminum oxide served as the positive control and negative control, respectively. (b) Cytotoxicity assay of alginate and AC using live and dead staining. Live cells stained with Calcein AM were presented as green while dead cells stained with EthD-1 were presented as red (Scale bar =100 *μ*m).

The cell viability of AC was assessed with the WST-1 assay. A medium with Al_2_O_3_ and ZDEC was used as the positive and negative control, respectively. There were no statistical differences in the cell viability between AC and control group as shown in Fig. 3(a). The results indicated the good biocompatibility of AC.

Cell toxicity was further evaluated by Live and dead staining with two kinds of fluorescent dyes (Calcein AM/EthD-1). Live and dead cells were shown in green and red, respectively, in Fig. 3(b). The results showed that cells treated with AC were still immobilized with normal morphology, indicating no cytotoxicity for AC.

### C. Barrier function test

**FIG. 4.**
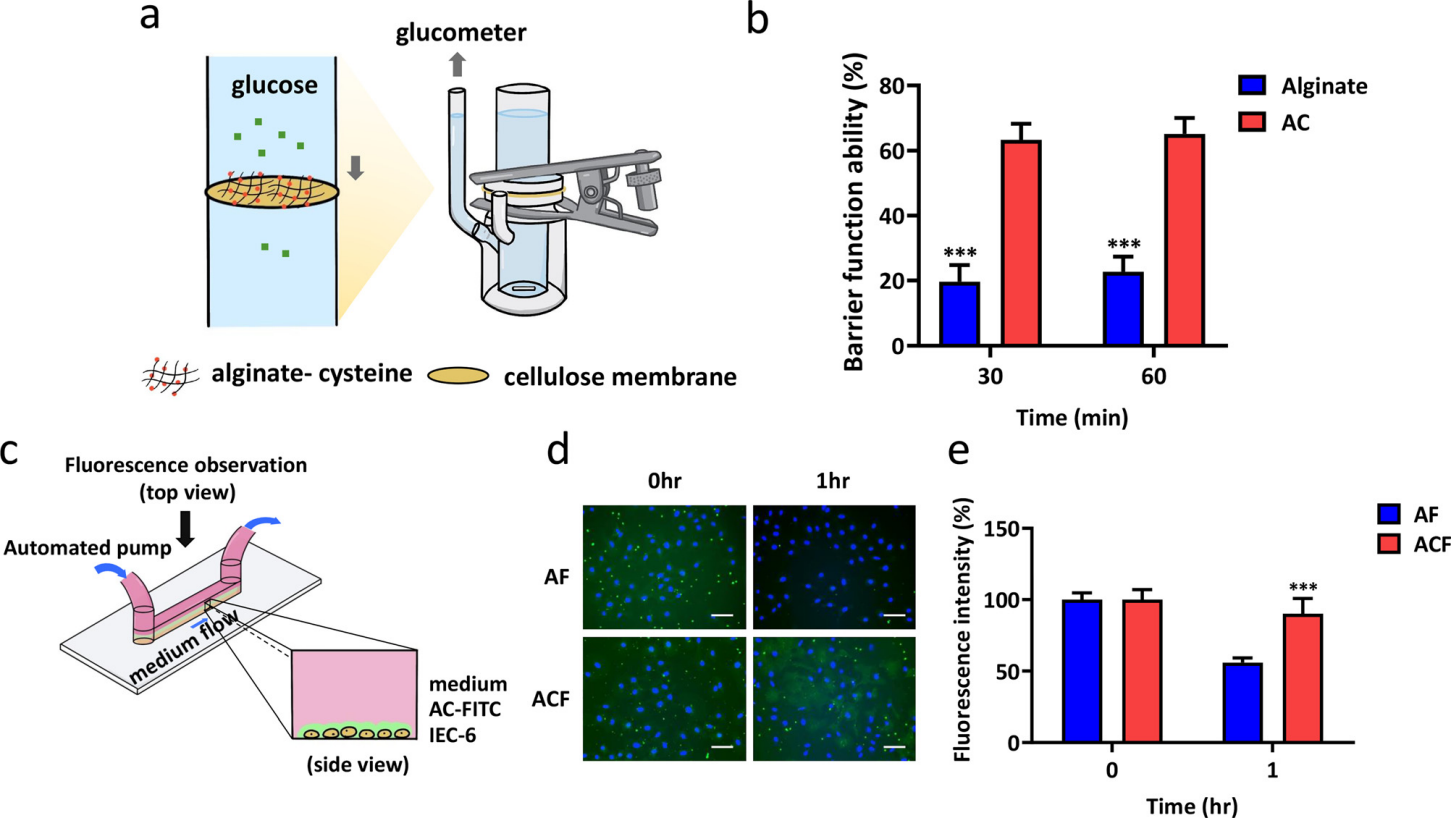
Barrier function and mucus adhesion test of AC *in vitro*. (a) Schematic representation of the barrier function test. 2 ml of the material was evenly applied on a mucin-coated cellulose membrane and fixed between chambers, and the glucose permeation was measured using a glucometer. (b) Relative barrier function ability of alginate and AC were tested with 500 mg/dl glucose. Two-way ANOVA with multiple comparisons (n = 3, ^***^P < 0.001 compared to alginate). (c) Schematic illustration of the *in vitro* mucus adhesion test. AF and ACF were incubated with IEC-6 in microfluid and applied constant flow of fluid. (d) Mucus adhesion test of alginate and AC under 1 h of the flow of medium and observed under the fluorescence microscope. AF and ACF were presented green, and the nucleus was presented blue in view (Scale bar =50 *μ*m) (e) Remaining fluorescence over time. Fluorescence at each time point was normalized to the initial fluorescence of AF and ACF (100%).Two-way ANOVA with multiple comparisons (n = 3, ^***^P < 0.001 compared to alginate).

To investigate the barrier function of alginate and AC, a mucin-coated membrane was used to mimic the mucus surface of the intestine [Fig. 4(a)]. The permeability of glucose through the membrane was utilized as the indication of barrier function. As shown in Fig. 4(b), the barrier property was about 63.4% in the AC group and about 19.6% in the alginate group, indicating a 44% better barrier function than alginate at the first 30 min. With time passing by, alginate inhibited only 22.7% of glucose permeation, and AC reduced over 65.1% of glucose permeation after 1 h. The results indicated the remarkable barrier properties of AC against nutrition absorption.

### D. *In vitro* mucus adhesion property

To evaluate the mucus adhesion of the material to the intestinal epithelium, FITC-labeled materials were incubated with IEC-6 cell line in a microslide, which was subjected to a constant medium flow by the syringe pump system for a period of time to simulate the shear stress in the gastrointestinal tracts [Fig. 4(c)]. As shown in Figs. 4(d) and 4(e), the fluorescent signals of alginate-FITC (AF) obviously decreased (44% reduction) but the fluorescence of alginate-cysteine-FITC (ACF) almost remained intact (10% reduction), thus indicating that covalently attaching cysteine to alginate could indeed increase the mucus adhesion.

### E. *In vivo* mucus adhesion property

**FIG. 5.**
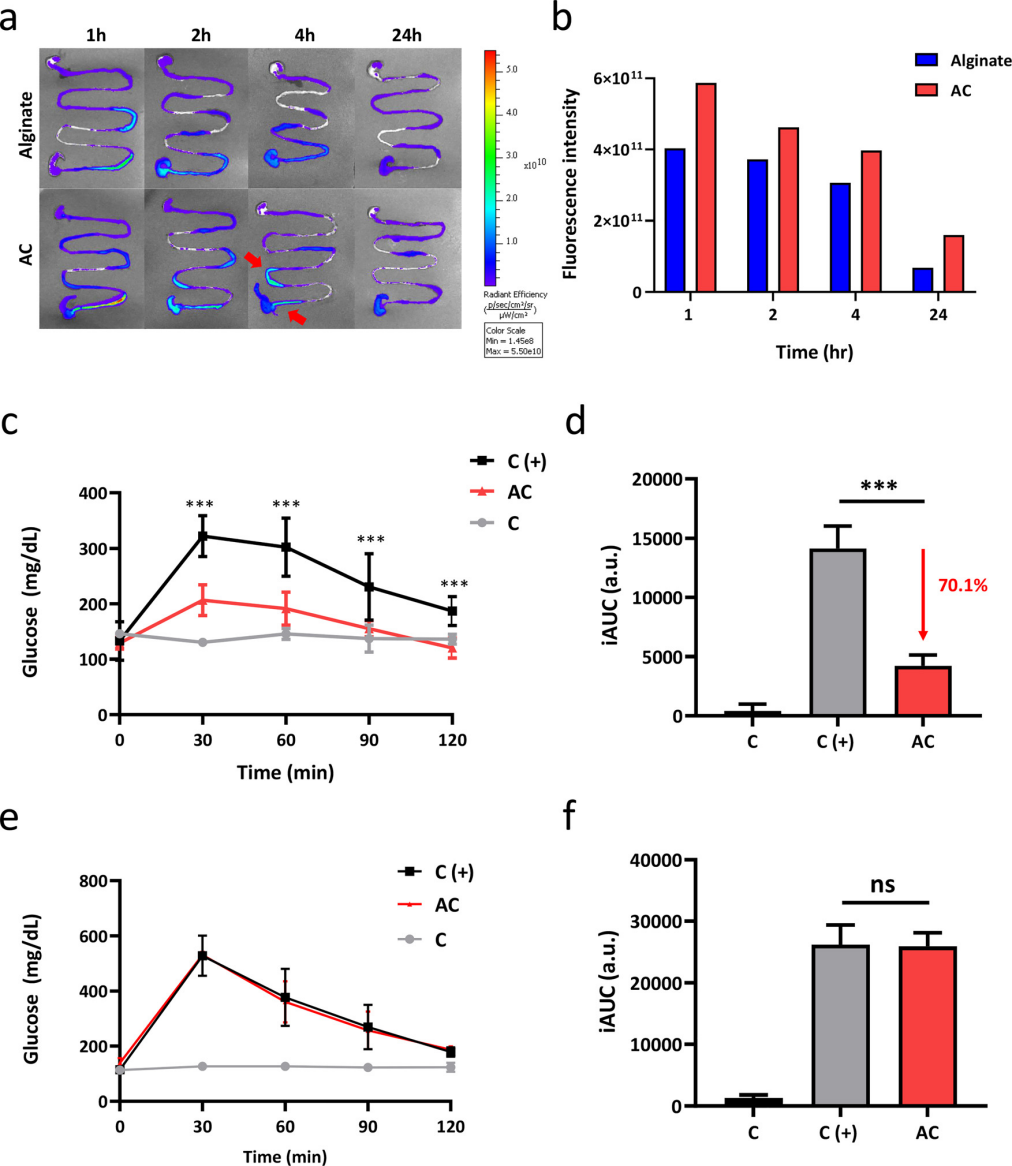
Evaluation of mucus adherence and glucose tolerance *in vivo* with AC administration in mice. (a) Mice were orally gavaged with 450 mg/kg materials encapsulated with FITC-albumin, and the gastrointestinal tracts from the stomach to cecum were harvested in 1, 2, 4, 24 h and imaged using an *in vivo* imaging system. Mice gavaged with PBS were used as control. (b) Fluorescence intensity of material at each time point (c) The glucose level of mice after oral administration of AC and glucose were recorded at each time point. Mice gavaged with PBS were used as control. Two-way ANOVA with multiple comparisons (n = 3, ^***^P < 0.001 compared to positive control). (d) iAUC of the OGTT curves. One-way ANOVA with multiple comparisons (n = 3 per arm, ^***^P < 0.001 compared to positive control). (e) The glucose level of mice after oral administration of AC and IP injection of glucose at each time point were recorded. Two-way ANOVA with multiple comparisons (n = 3 per arm). (f) iAUC of the IPGTT curves. One-way ANOVA with multiple comparisons (n = 3 per arm).

To further assess the ability of AC to form a coating *in vivo*, C57BL/6 mice were gavaged with fluorescence encapsulated polymers. The fluorescent particle (FITC-albumin) not only provided the signals to trace the materials but simulated potential drugs at the same time. After oral administration of materials, the gastrointestinal tracts were harvested at indicated time points and measured under IVIS [Fig. 5(a)]. After 1 h, fluorescent signals were observed in the duodenum and intestine for both the alginate and AC group, while it was much intense for the AC group. As time passed by, the signal of alginate quickly reduced. On the other hand, much more fluorescence was detectable for the AC group.

As for fluorescence quantification, the signal intensity was lower for the alginate group than AC at each time point [Fig. 5(b)]. The signals remained after 24 h, revealing the excellent mucus adhesion of AC, which corresponded to the results of the *in vitro* adhesion test and indicated a sustained release of particles. With time passing, the intensity of signals decreased, indicating that AC formed a temporary coating on the gastrointestinal tracts and would be discharged under metabolism without causing burden to the body.

### F. Evaluation of the effect of AC on glucose tolerance

An oral glucose tolerance test (OGTT) was performed to evaluate the barrier effect of AC on nutrition, and glucose was selected as the representative molecule. In the OGTT, the plasma glucose increased to a maximum after 30 min of oral administration of glucose in both the control and AC group, but the peak value was much lower in the AC group [Fig. 5(c)]. Compared to the control group, the absorption of glucose was reduced by 70.1% in the AC group [Fig. 5(d)]. To further investigate whether the decreased glucose response is owing to a systematic effect or not, IPGTT was performed. The glucose level of both the control group and the AC group rose to around 530 mg/dl and exhibited a dramatic decline after the first 30 min [Fig. 5(e)]. In addition, there were no statistical differences of AUC between the AC and control group, indicating the reduction of glucose response was not due to a systematic effect [Fig. 5(f)]. Above all, we assumed that AC could effectively bind to mucin of the surface intestine and form a physical barrier to reduce adsorption.

### G. Release profile of resveratrol and ACR

**FIG. 6.**
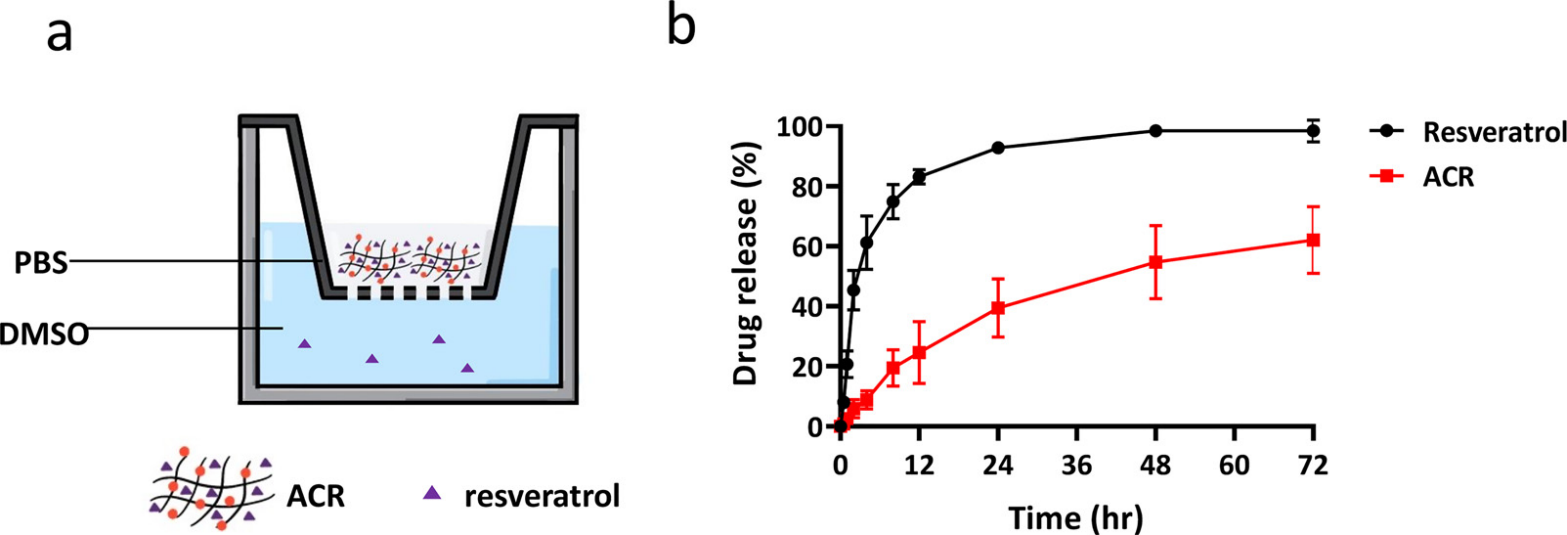
Release profile of Resveratrol and ACR. (a) Schematic of transwell system for the drug release evaluation. (b) Release profile of ACR and resveratrol only against DMSO with the transwell system at room temperature.

The transwell system was used to evaluate the release profile of resveratrol with or without the AC carrier [Fig. 6(a)]. The results showed in Fig. 6(b) was a two-stage release. We hypothesized that a portion of resveratrol adhered to the surface of AC, which rapidly diffused in the first 8 h. As time passed by, the release rate went down and the reaction achieved a balanced state. The two groups showed obvious differences after 24 h, with over 90% of resveratrol without carrier released in 24 h, but only 40% of resveratrol released with the AC carrier. The results indicated that AC was a potent controlled-release carrier for resveratrol.

### H. Evaluation of the effect of ACR on liver damage

**FIG. 7.**
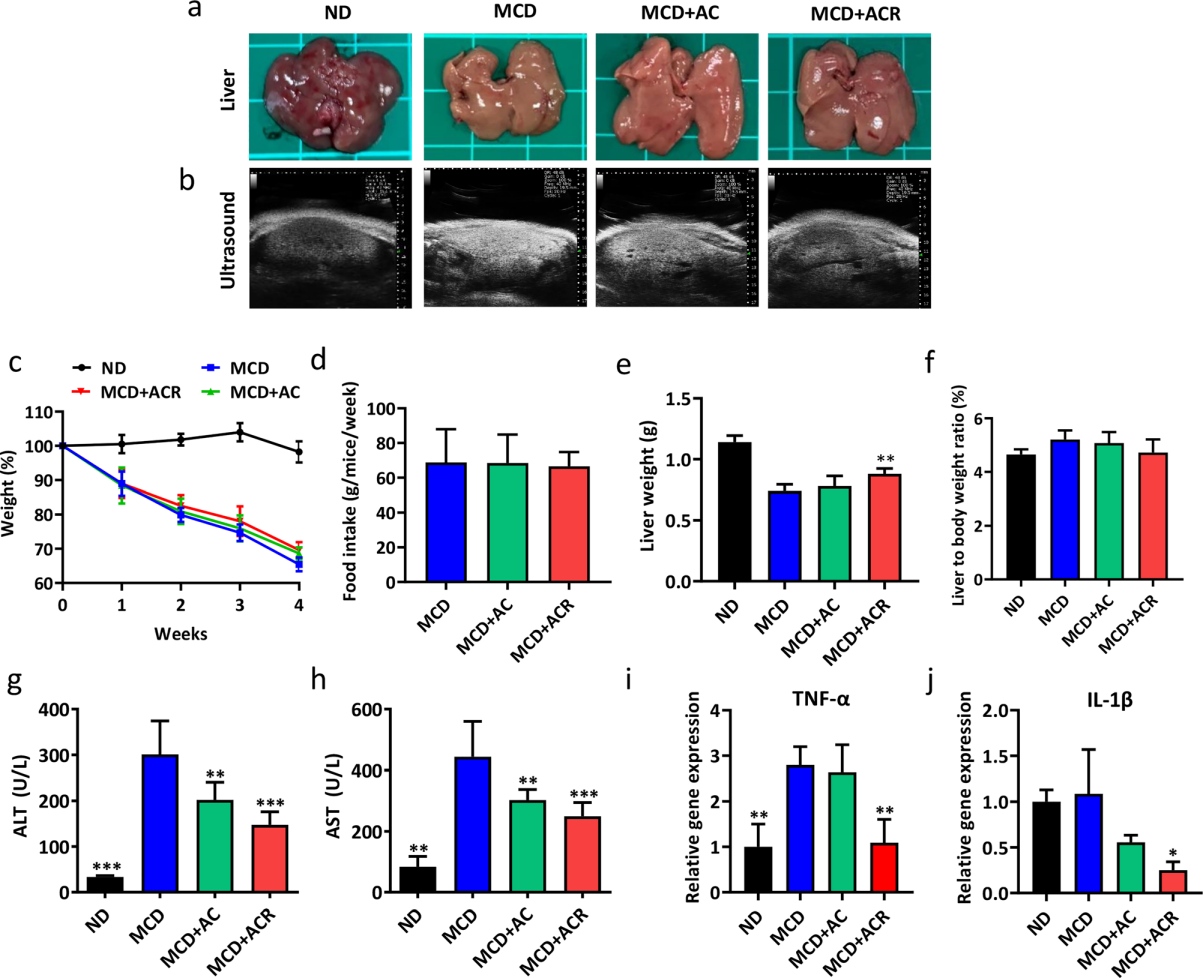
The effect of ACR on alleviating hepatocellular damage in MCD-fed mice. (a) Representative images of the liver and (b) liver ultrasound images of mice, (c) mice fed with MCD diet were given oral administration of saline, AC, or ACR for four weeks; Mice fed with normal diet were used as the control. The weight of mice normalized to 0 week of each group was shown. (d) Intake of MCD diet of each group, (e) liver weight, and (f) liver to body weight ratio were evaluated after sacrifice, one-way ANOVA with multiple comparisons (n = 5 per arm, ^**^P < 0.01 compared to MCD). Serum level of (g) ALT and (h) AST, one-way ANOVA with multiple comparisons (n = 5 per arm, ^**^P < 0.01, ^***^P < 0.001 compared to MCD). Relative expression of (i) TNF-α and (j) IL-1β, one-way ANOVA with multiple comparisons (n = 3 per arm, ^*^P < 0.05, ^**^P < 0.01, and ^***^P < 0.001 compared to MCD).

To investigate the potential of AC to ameliorate NAFLD, a methionine choline-deficient (MCD) diet was used to induce fatty liver disease. C57BL/6 mice were fed with MCD diet and orally gavaged with saline, AC, or ACR daily, and the control group was fed with normal diet (ND) and gavaged with saline. Gross observation of the livers from saline-treated MCD diet-fed mice appeared paler due to the accumulation of hepatic lipids, AC treated MCD diet-fed mice appeared less pale, whereas the ACR treated MCD diet-fed mice showed a reddish color similar to the normal diet group [Fig. 7(a)]. The ultrasound images of the MCD group showed a bright and blurry view, indicating the scattering effects by lipid droplets of steatosis, although the image of the AC group was similar to the MCD group, while the shades of the ACR group image was similar to the image of ND group [Figs. 7(b) and S2]. Furthermore, it was obvious that MCD diet feeding dramatically cause weight loss, while mice treated with AC and ACR exhibited an improved situation [Fig. 7(c)]. The average food intake showed no significant differences between these groups, thus excluding the possibility of food consumption to affect the results [Fig. 7(d)]. ACR treatment also increased the liver mass and decreased the liver to body weight ratio compared to the MCD diet group [Figs. 7(e) and 7(f)].

The serum level of alanine aminotransferase (ALT) and aspartate aminotransferase (AST) was significantly higher in the MCD diet-fed mice compared to the normal diet. However, the levels were significantly lower in AC and ACR group compared to the MCD group. The results indicated that AC and ACR treatment could attenuate hepatocyte cell death on the MCD diet [Figs. 7(g) and 7(h)]. To evaluate the inflammation triggered by the MCD diet, mRNA expression levels in the liver were assessed by qPCR. Consistent with the results of serum biochemistry and histological analysis, the mRNA expression of TNF-α and IL-1β was increased in the MCD group and decreased when treated with AC and ACR [Figs. 7(i) and 7(j)]. The results indicated that AC and ACR treatment reduced the inflammatory response triggered by the MCD diet. The biosafety of ACR was further confirmed by the serum level of blood urea nitrogen (BUN), creatinine (CRE) (Fig. S3), and the complete blood test (Table S2).

### I. Evaluation of the reduction in lipid accumulation with ACR

**FIG. 8.**
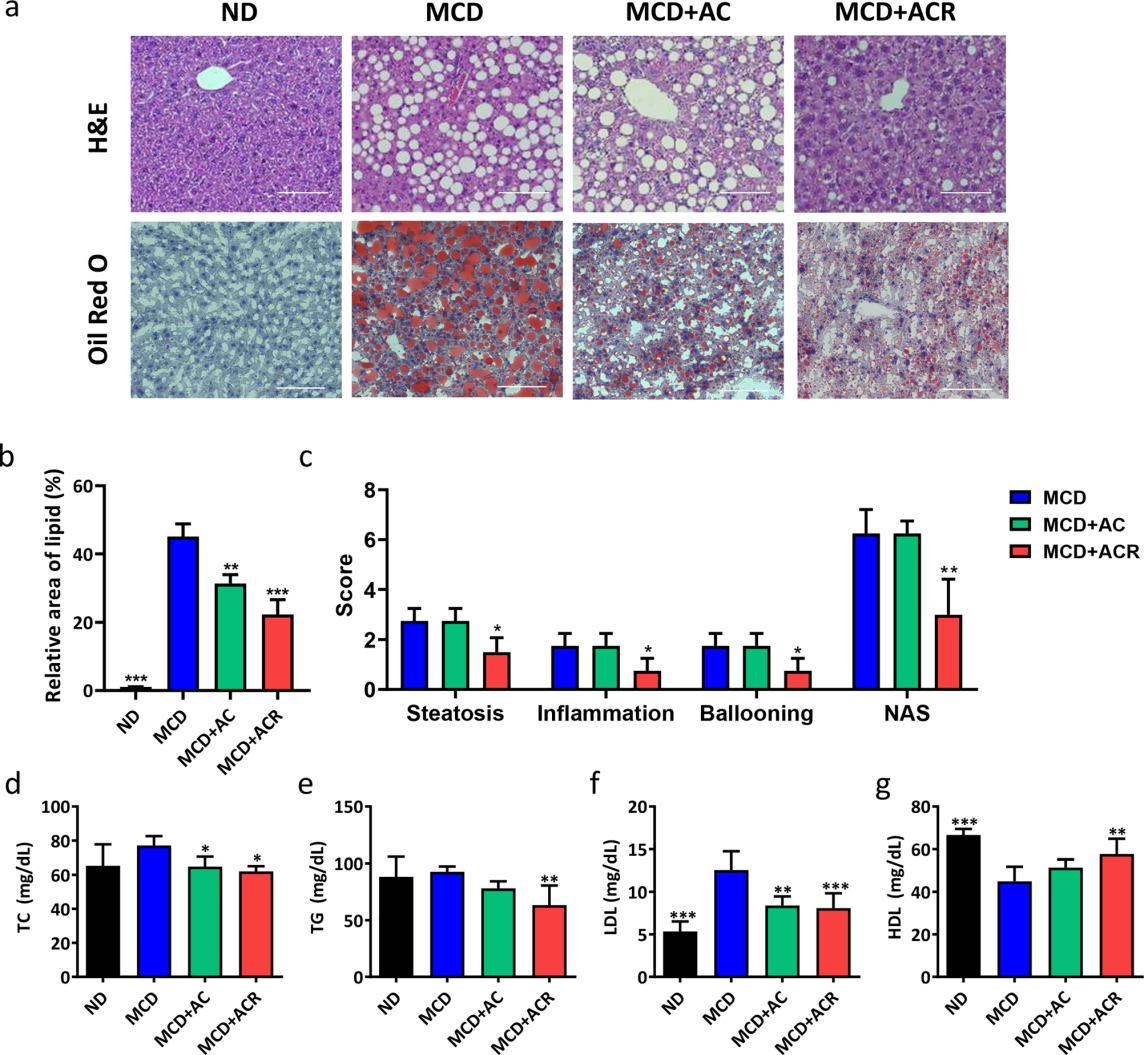
The effect of ACR on attenuating hepatic steatosis in MCD induced mice. (a) H&E stain and oil red o stain of liver sections, (b) quantification of the positive signal area from oil red o stain of liver sections, one-way ANOVA with multiple comparisons (n = 3 per arm, ^*^P < 0.05, ^**^P < 0.01, and ^***^P < 0.001 compared to MCD). (c) Score of steatosis, inflammation, ballooning, and NAFLD activity score (n = 4 per arm, ^*^P < 0.05, ^**^P < 0.01 compared to MCD) (d)–(g) serum level of TC, TG, LDL, and HDL, one-way ANOVA with multiple comparisons (n = 5 per arm, ^*^P < 0.05, ^**^P < 0.01, and ^***^P < 0.001 compared to MCD).

The histological and biochemical analysis determined the pathogenesis of NAFLD. The results of HE staining indicated that the structure of the cell was normal and with no fatty degeneration in the normal diet group, while lipid degeneration was observed in the MCD group with the cells swelling. Importantly, the hepatocyte fatty degeneration in AC and ACR group was decreased compared to the MCD diet group, particularly obvious in the ACR group [Fig. 8(a)]. Consistent with H&E staining, the results of oil red O staining showed that the lipid droplets significantly increased in the MCD group compared to those in the ND group, while the ACR treatment reduced about 23% of the lipid droplets compared to the MCD group [Figs. 8(a) and 8(b)]. Additionally, the NAFLD activity score (NAS) was utilized to evaluate the liver tissues. The NAS score of the ACR group was much lower than MCD and AC group. As for the histological score for steatosis, inflammation and ballooning were mostly high in the MCD group and AC group. However, all of the scores were decreased in the ACR group and with significant differences compared with the MCD group [Fig. 8(c)]. Based on H&E and Oil Red O staining, NAS for steatosis demonstrated that the liver pathology of mice induced by MCD was significantly attenuated by ACR. To assess the evolution of steatohepatitis, a series of the metabolic parameter was provided. The results indicated that the MCD diet caused serum levels of TC, TG, and LDL to increase, but all decreased with ACR treatment. The serum level of HDL was decreased with the MCD diet but increased with ACR treatment [Figs. 8(d)–8(g)]. The biosafety of AC and ACR were also ensured with complete blood count (Table S2).

## III. DISCUSSION

NAFLD is recognized as excess lipid accumulation in the liver owing to an unhealthy lifestyle, genetic factors, or metabolism factor. The spectrum of liver damage progresses from simple steatosis, steatohepatitis, cirrhosis to carcinoma. To slow down the progression of NAFLD, it is highly suggested to modify lifestyle with energy restriction in the early stage, improve hepatic steatosis in the absence of medical side effects and surgery complications, and avoid advanced irreversible liver damage.

Recently, herbs have gained interest to be a beneficial therapeutic approach for patients with NAFLD due to their antioxidant and anti-inflammation effect.^21^ Resveratrol (3,5,4′-trihydroxystilbene), a natural polyphenol, is one of the popular candidates. The results of research implied that it might regulate hepatic lipid metabolism and the clearance of lipid accumulation through the AMPKα-SITR1 pathway or autography.^30–32^ However, resveratrol exhibits limitations in clinical trials due to its poor availability and low bio-efficacy, which attributes to extensive and rapid metabolism.^27,33^

To solve the problem, researchers focus on developing a drug delivery system to improve the pharmaceutical properties of resveratrol. Carriers such as liposome, micelle, polymer, and nanoparticle have been evaluated.^34,35^ Moreover, some researchers point out the possibility to enhance the efficacy of carriers leveraging their mucoadhesive properties to the intestinal mucosa.^36^ The adherence of polymers to a mucosal membrane involved different interactions, such as covalent linkage, electronic attraction, surface, and interfacial energy.^37^ Among them, the formation of covalent linkage exerts significant performance. For example, the adherence of thiolated polymer can adhere better to mucin via forming a disulfide bond.^18^ Within the gastrointestinal tract, the major digestive part was located in the intestine, containing consistent epithelial cells and globlet cells, which produce the mucus layer. The main composition of the mucus layer was mucin, one of the glycoproteins, with a highly glycosylated central region, cysteine-rich domain, and branched carbohydrate component. Some redox couples participate in intestinal redox systems, such as glutathione, thioredoxin, and cysteine to mediate free radical attack. They help maintain the redox environment, mucosal antioxidant defense, and most importantly, provide enough free thiol groups for thiolated materials to form covalent linkages and adhere. In addition, these redox pairs are in a dynamic equilibrium state.^17,38^ Therefore, the thiolated materials would be excreted out of the body with peristalsis, thereby eliminating the toxicity concern and the accumulation of reactive molecules.

In this study, AC was obtained with the formation of amide bond between the amino group of cysteine and the carboxyl group of alginate with EDC and NHS reaction. EDC/NHS is a widely used crosslinker in the biochemical field with its water-soluble property.

In the FTIR spectrum, the peak of the thiol group attributed from conjugated cysteine was shown between 2550 and 2660 cm^−1^, while the peak was not obvious. We hypothesized that it resulted from the weak signal intensity of thiol itself, and it might also be affected by the strong and sharp peak of OH stretching nearby [Fig. 2(b)]. Ellman's reagent is a common method for examining thiol content owing to the specificity for the thiol group and ease of measurement. The product appears in visible yellow color as the reagent reacted with the thiol component and can be detected under 420 nm.^39^ The result indicated the degree of thiol group substitution on alginate was about 10% [Fig. 2(c)]. The chemical structure of AC was also identified by NMR, and the cysteine signals can be identified at 3.2 and 4.2 ppm via comparing the spectrometry to the original alginate, while the signal of NMR around 2–3.6 ppm was assumed to be caused by excessive crosslinkers, EDC and NHS [Fig. 2(d)].

In the barrier function test, glucose was chosen as it is the main energy source of animals, and the result of the barrier function test indicated that AC could block more glucose permeation [Fig. 4(b)]. Moreover, it was shown that the rapid change of glucose level after meals is highly related to metabolic syndrome. Judging from the results, the glucose level of the mice orally administrated with alginate is elevated higher than the AC group at each time point, and the glucose level of the mice orally administrated with AC was closer to the control group. As for the IPGTT test, there are no obvious differences between the alginate and AC group. According to the results, we hypothesized that the influence of reduced absorption was due to the formation of a physical barrier instead of a systematic effect [Figs. 5(c)–5(f)]. Therefore, we believed that AC demonstrated the possibility to treat metabolic syndromes such as diabetes and obesity. From the long-term evaluation, reduced serum level of TC, TG, and LDL as well as elevated serum level of HDL with ACR treatment also support our hypothesis that AC collaboratively contributes to the beneficial effects on metabolic parameters with resveratrol involved [Figs. 8(d)–8(g)].

To mimic the intestinal environment, the syringe pumping rate is in accordance with the shear force produced during intestinal peristalsis as 1.8 *μ*N cm^−1^.^16^ The result *in vitro* showed that the mucoadhesive property of AC is better than alginate under such conditions with intestinal epithelioid cell line, IEC-6 [Figs. 4(d) and 4(e)]. Albumin-FITC was a common fluorescent compound commonly used in tracking;^40^ therefore, it was selected as the fluorescent signal and be capsuled in the materials to mimic the release of drugs *in vivo*. The result of IVIS showed that the adhesion of the material to mucin was enhanced via modification with the thiol group. After 1 h, the still-intense signal of AC suggested that it could retain much longer beyond the course of two meals daily [Figs. 5(a) and 5(b)].

To investigate the therapeutic effect of ACR, MCD diet was selected to induce NAFLD. MCD diet-induced model is well recognized for the study of steatosis and steatohepatitis. As the precursors of phosphatidylcholine, methionine and choline play an important role in TG export through very low-density lipoproteins (VLDL) packaging. The deficiency of methionine and choline makes an influence on VLDL production or secretion and the export of TG.^41,42^ In this study, AC from the physical barrier makes the reduction in excessive nutrition absorption, which lead to weight loss. However, MCD diet also causes weight loss, indicating the limitation of AC on distinguishing the therapeutic effect on weight loss. Therefore, histological stain and the serum level of metabolism factors would be better indicators. A high-fat diet model would be another choice for the investigation of decreased nutrition absorption in the future. H&E staining indicated that the hepatic lobules of the normal mice were complete, with a clear view of the central vein and hepatic sinus. On the other hand, it showed swollen hepatocytes and fatty degeneration in the MCD diet group, demonstrating liver damage. Importantly, the hepatocyte fatty degeneration decreased and the liver damage was less severe in AC and ACR group compared to that in the MCD group [Fig. 8(a)]. In addition, the serum levels of ALT and AST were common indicators for liver damage and metabolism syndrome.^43–45^ The result of the serum test indicates the ability of ACR to decrease the ALT and AST levels [Figs. 7(g) and 7(h)]. Furthermore, members of the interleukin-1 (IL1) superfamily are thought to be involved in the inflammation processes such as obesity and liver disease. Among the cytokines, IL-1β contributes to the pro-inflammatory effects.^46^ Another common cytokine, TNF-α, produces the inflammatory mediators and cell death, involved in the pathophysiology of NAFLD.^47^ The results of qPCR indicated that the relative expression of TNF-α and IL-1β was lower by 2.57-fold and 4.34-fold, respectively, with ACR treatment compared to MCD. Compared to the AC group, the addition of resveratrol reduced 2.4-fold and 2.2-fold expression of TNF-α and IL-1β, respectively [Figs. 7(i) and 7(j)]. These results showcased the desirable performance of ACR on anti-inflammation and alleviating hepatic damage.

Above all, these results indicate that the developed thiolated alginate could be an effective mucoadhesive polymer for the administration of resveratrol as an alternative treatment for alleviating NAFLD with biosafety (Fig. S3 and Table S2). The developed ACR could be safe and easy to take the supplement as oral administration for the daily protection of liver steatosis. It also shows the potential for improvement of metabolic syndrome such as diabetes with its reduced response to glucose level.

## IV. CONCLUSION

In this study, we synthesized thiolated alginate by cross-linking alginate and cysteine using EDC/NHS. The developed AC formed a physical barrier in the intestine and reduced 70% of blood glucose response. The enhanced barrier function, mucoadhesive property, and non-cytotoxicity were investigated *in vitro* and *in vivo*. Moreover, AC could sustain for up to 24 h in the gastrointestinal tracts. In addition, AC showed promising carrier properties of resveratrol by promoting the extension time, thereby enhancing its bioavailability. In addition, the fat degeneration in liver and fat-related metabolic indicators in serum was significantly improved in the ACR treatment group without damaging the liver and kidney. In sum, these results indicated that the developed ACR is a safe mucoadhesive polymer, which made remarkable improvements in hepatic steatosis, showing the potential of being an oral administrated daily supplement to alleviate NAFLD.

## V. METHODS

With the concept of thiol group conjugation, synthesis and characterization of alginate-cysteine were evaluated, which is followed by the barrier function ability test. The cytotoxicity and carrier property were investigated with the release profile, WST-1 and live and dead stain. The mucoadhesive property was also evaluated both *in vitro* and *in vivo*. Finally, the long-term therapeutic effect of ACR was evaluated *in vivo*.

### A. Synthesis of alginate-cysteine (AC)

Alginate was chemically modified utilizing aqueous carbodiimide chemistry. The carboxylic acid groups of alginate were activated by the addition of N-(3-dimethylaminopropyl)-N-ethylcarbodiimide (EDC). To avoid rapid hydrolysis of the reactive EDC-mediated coupling reaction, N-hydroxysuccinimide (NHS) was added to stabilize the coupling reaction. 1 g of sodium alginate (Sigma-Aldrich) was dissolved in 25 ml of 0.1M MES buffer (pH 5.5, Sigma-Aldrich). Then, the same volume of EDC (Sigma-Aldrich) and NHS (Fluka) solution was added to the final concentration of 100 mM, respectively, and pH was adjusted to 6.0 using 5M NaOH solution. After 90 min of carboxylic group activation, L-cysteine hydrochloride (Sigma-Aldrich) was added to a mole ratio of 1:4 (alginate:L-cysteine hydrochloride) and further reacted for 20 h at pH 6.0. The whole reaction proceeded at room temperature and exposure to light was avoided. To remove the unreacted EDC, NHS, and L-cysteine hydrochloride, the product was dialyzed against 1 mM HCl aqueous solution at room temperature with a dialysis membrane (MWCO:6000–8000 Da). The pH of the sample was adjusted to 7.0 before lyophilization and stored at room temperature for subsequent analysis.

### B. Structure characterization

Fourier Transform Infrared (FTIR) spectroscopy was acquired on spotlight 200i with the auto-attenuated total reflection (ATR) system of Perkin Elmer. Spectra with wavelengths of 400–4000 cm^−1^ were obtained at a resolution of 4 cm^−1^.

Microscopic imaging analysis was conducted with a JEOL JSM-6510 SEM. Freeze-dried alginate and AC were placed on a platinum plate with an electrically conductive carbon tape. Samples were coated with gold for 60 s with a current of 30 mA. Elemental analysis of samples was evaluated with energy-dispersive x-ray spectroscopy (EDS). The results showed the percentage of carbon, hydrogen, and thiol of alginate and AC, demonstrated at atomic percentage.

1H NMR spectra were acquired on a Bruker Avance III 500 spectrometer operating at 500 MHz in a magnetic field of 11.75 T at ambient temperature. The NMR experiment was prepared with alginate and AC dissolved in D_2_O to a final concentration of 10 mg/ml (mg/ml or mg ml-1depends on journal guidelines).

The degree of thiolation was determined by Ellman's assay. Ellman's reagent (5,5-dithio-bis-2-nitrobenzoic acid or DTNB) produces yellow-colored products as it reacts with the sulfhydryl group. Briefly, an equal volume of 2 mg/ml AC in phosphate buffer (pH) was mixed with the Ellman's reagent and reacted in the dark at 37 °C for 2 h, followed by the absorbance measurement at 420 nm with a plate reader.

### C. Barrier property

To investigate the barrier property of candidate materials, a mucin-coated membrane was prepared to mimic the mucus surface of the intestine. A fixed concentration of mucin (Sigma-Aldrich) was used to achieve reproducible results. A cellulose membrane (pore size 6 *μ*m, Advantec) was incubated in 3% w/v porcine stomach mucin in ddH_2_O and gently shaken for 1 h at room temperature. After that, 2 ml of candidate material was applied evenly to cover the whole mucin-coated membrane and rested for one more hour. The material-coated membrane was fixed between the chambers of the Franz cell system, 20 ml of 500 mg/dl glucose was added gently in the upper chamber against ddH_2_O in the lower chamber with a stir. 1 ml of the sample was collected from the lower chamber at 30 and 60 min, and the glucose concentration was measured using a glucometer (Accu-Chek Instant, Roche). The barrier property tests were repeated three times individually for each material.

### D. Release profile of ACR

The release profile was evaluated by the transwell system. It is a common way to evaluate the drug release profile as resveratrol gradually goes through the permeable membrane. Alginate-cysteine with resveratrol (ACR) was placed in the transwell insert (0.8 *μ*m pore size) against DMSO, and 100 *μ*l of the outer DMSO was collected followed by the addition of another 100 *μ*l of fresh DMSO at 0, 0.25, 0.5, 1, 2, 4, 8, 12, 24, 48, 72 h. The absorbance of resveratrol was measured at 266 nm with a reader plate and repeated three times.

### E. Cytotoxicity

Cell viability was investigated using WST-1 assay. L929 cells were seeded in 96 well culture plates with a density of 10 000 cells per well and incubated for one day until full adhesion. 0.2 g/ml alginate, alginate-cysteine (AC), Al_2_O_3_, and ZDEC were dissolved in Minimum Essential Medium (MEM) as extraction fluids for one day. Then, the cell culture medium was replaced with the extraction of materials. After 24 h of incubation, the medium was discarded and replaced with the WST-1 assay reagent and incubated at 37 °C for 2 h. The absorbance was read at 450 nm with a plate reader.

Live and dead staining was employed to evaluate the cytotoxicity of materials. Briefly, L929 cells were seeded with a density of 30 000 cells per well in a 12 well culture plate for one day until full adhesion. Alginate and AC were dissolved in MEM as extraction fluid for one day, and the cell culture medium was replaced with the extraction of materials. After 24 h of incubation, triton was added before cell staining as the positive control. Calcein-AM and ethidium homodimer-1 (EthD-1) were used to stain living and dead cells, respectively. After 30 min of incubation, the results were imaged under a fluorescence microscope.

### F. *In vitro* mucus adhesion test

Mucus adhesive properties were evaluated using a *μ*–Slide syringe pump system. The fluorescence-labeled materials were synthesized with FITC via the EDC/NHS modification. First, 30 000 IEC-6 cells (Intestinal epithelioid cells) were seeded in a commercial microfluidic channel (ibidi *μ*-Slide I 0.4 Luer). After overnight incubation, the nucleus was stained with Hoechst 33342 for 30 min. Then, 2% of alginate-FITC (AF) and alginate-cysteine-FITC (ACF) (pH was adjusted to 7) were incubated with cells at 37 °C for 1 h to allow the interaction of materials and cells. The microfluidic device was then subjected to a constant flow of DMEM with a syringe pump at a flow rate of 150 *μ*l/min for 1 h. The remaining fluorescence was observed under a fluorescence microscope at 0 and 60 min.

### G. Animals

c57BL/6 male mice (6–8 weeks old) purchased from Lasco were housed in five at a controlled temperature and 12 h dark-light cycle (lights on at 8 a.m.). Mice were acclimatized for one week before experiments. All the mice were free to diet and water during experiments. The study protocol was approved by the Institutional Animal Care and Use Committee of the National Taiwan University (IACUC, No. NTU-20201062).

### H. Oral glucose tolerance test (OGTT) and Intraperitoneal glucose tolerance test (IPGTT)

To investigate the effect of alginate and AC *in vivo* postprandial glucose absorption or systemic absorption, mice were pregavage with the materials, and OGTT and IPGTT were adopted subsequently. After fasting for 20 h, mice were orally gavaged with 250 mg/kg of alginate and AC, respectively, and the positive and negative control groups were administered with only PBS. Then, 4 g/kg mouse of glucose were administered 1 h later, except that the negative control group was administered with PBS only. The blood was collected from the tail vein and the glucose concentration was measured with a glucometer (Accu-Chek Instant, Roche) every 30 min until the blood glucose level was stable at 120 min.

### I. Fluorescent imaging of the GI tract with AC gavage

To investigate the adherence property of AC *in vivo*, C57BL/6 mice were orally gavaged with 250 mg/kg of alginate or AC encapsulated with 10% FITC-BSA (w/w). The gastrointestinal tracts from the stomach to cecum were harvested after 1, 2, or 4 h and imaged using an IVIS Luminar II *in vivo* imaging system (Perkin Elmer). Mice without material gavage were used as control, and all images were normalized to control.

### J. Long-term treatment of AC

Mice were randomly divided into four groups: normal diet (ND), methionine choline-deficient (MCD) diet, MCD diet with AC (MCD+AC), and MCD diet with AC and resveratrol (MCD+ACR). ND and MCD groups were orally gavaged with saline solution daily. The AC group was orally administered with 250 mg/kg of AC, and the MCD+ACR group was treated with 250 mg/kg AC encapsulated with 20 mg/kg of resveratrol. After 4 weeks of treatment, the blood was collected via cardiac puncture and centrifuged at 10 000 g for 10 min at 4 °C to obtain the serum. Harvested livers were weighed and either frozen (optimal cutting temperature compound, OCT) for Oil Red O staining or fixed in 10% formaldehyde for hematoxylin-eosin (H&E) staining.

### K. Ultrasound imaging

The hepatic steatosis was evaluated with ultrasound imaging at the end of the experimental period. c57BL/6 mice were anesthetized with isoflurane (%) and placed on the observation plate. The liver was imaged with a transductor using Prospect Ultrasound Imaging System. B-mode imaging of the livers was recorded with a 48 dB dynamic range and 40 MHz frequency in a large lateral view. The semiquantitative analysis was done in the relatively same area of the liver among the groups using image J software.

### L. Quantitative PCR (qPCR)

After sacrifice, livers removed from mice were washed in saline and quickly frozen in liquid nitrogen, kept at −80 °C. Total RNA was extracted from the liver using TRIzol following the protocol of Direct-zol™ RNA MiniPrep Kits (Zymo Research, Irvine, CA, USA). RNA was reverse-transcribed to acquire cDNA using Super-Script^®^ IV Reverse Transcriptase (Thermo Fisher Scientific). For qPCR analysis, cDNA was mixed with primers and SYBR Green Master Mix (Thermo Fisher Scientific). The intensity was detected and recorded by LightCycler^®^ 480 Instrument (Roche Diagnostics Nederland BV, The Netherlands). Relative expression levels of IL-6 and TNF-α genes were normalized with the GAPDH housekeeping gene and calculated using the delta-delta Ct method.

### M. Statistics

The experimental results were shown as mean with at least three replicates. The statistical differences analysis adopted the one-way ANOVA with Tukey's multiple comparisons and two-way ANOVA with Sidak's multiple comparisons. Differences were considered significant at a P-value of less than 0.05 (P < 0.05, ^*^; P < 0.01, ^**^; P < 0.001, ^***^).

## SUPPLEMENTARY MATERIAL

See the supplementary material for SEM image of alginate and AC; quantification of the liver ultrasound image of mice fed with normal diet and saline treated; MCD diet with saline, AC, and ACR treated separately; and evaluation of kidney function with the serum level of creatinine and blood urea nitrogen. Tables include the elemental analysis of alginate and AC and safety evaluation with complete blood count test.

## Data Availability

The data that support the findings of this study are available within the article and its supplementary material.
